# Preliminary evaluation of the antiglycoxidant activity of verapamil using various *in vitro* and *in silico* biochemical/biophysical methods

**DOI:** 10.3389/fphar.2023.1293295

**Published:** 2023-11-28

**Authors:** Miłosz Nesterowicz, Kamil Klaudiusz Lauko, Karolina Dańkowska, Daria Trocka, Małgorzata Żendzian-Piotrowska, Jerzy Robert Ładny, Anna Zalewska, Mateusz Maciejczyk

**Affiliations:** ^1^ Students’ Scientific Club “Biochemistry of Civilization Diseases” at the Department of Hygiene, Epidemiology and Ergonomics, Medical University of Bialystok, Bialystok, Poland; ^2^ Department of Hygiene, Epidemiology and Ergonomics, Medical University of Bialystok, Bialystok, Poland; ^3^ Department of Emergency Medicine, Medical University of Bialystok, Bialystok, Poland; ^4^ Independent Laboratory of Experimental Dentistry, Medical University of Bialystok, Bialystok, Poland

**Keywords:** verapamil, diabetes mellitus, protein glycation, antiglycative activity, antioxidant activity

## Abstract

**Introduction:** Glycoxidative stress is essential for linking glucose disturbances and cardiovascular diseases. Unfortunately, contemporary antidiabetic drugs do not have an antiglycative effect but only lower blood glucose levels. Therefore, there is an intense search for substances that could inhibit protein glycation and prevent diabetic complications. A potential antioxidant activity has been demonstrated with verapamil, a phenylalkylamine derivative belonging to selective calcium channel blockers. Verapamil has a well-established position in cardiology due to its wide range of indications and good safety profile. Nevertheless, the antidiabetic activity of verapamil is still unclear. We are the first to comprehensively evaluate the verapamil’s effect on protein glycoxidation using various *in vitro* and *in silico* models.

**Methods:** Bovine serum albumin (BSA) was used to assess the rate of glycoxidation inhibition by verapamil. As glycating factors, sugars (glucose, fructose, and ribose) and aldehyde (glyoxal) were used. Chloramine T was used as an oxidizing agent. Aminoguanidine (protein glycation inhibitor) and Trolox (antioxidant) were used as control substances. The biomarkers of oxidation (total thiols, protein carbonyls, advanced oxidation protein products), glycation (Amadori products, β-amyloid, advanced glycation end products [AGEs]), and glycoxidation (tryptophan, kynurenine, N-formylkynurenine, dityrosine) were evaluated using colorimetric and fluorimetric methods. The mechanism of antiglycative activity of verapamil was assessed using *in silico* docking to study its interaction with BSA, glycosidases, and seventeen AGE pathway proteins.

**Results:** In all *in vitro* models, biomarkers of protein glycation, oxidation, and glycoxidation were significantly ameliorated under the influence of verapamil. The glycoxidation inhibition rate by verapamil is comparable to that of potent antiglycating agents and antioxidants. The molecular docking simulations showed that verapamil bound preferentially to amino acids prone to glycoxidative damage out of an α-glucosidase’s active center. Among all AGE pathway proteins, verapamil was best docked with the Janus kinase 2 (JAK2) and nuclear factor-κB (NF-κB).

**Discussion:** The results of our study confirm the antiglycoxidant properties of verapamil. The drug’s action is comparable to recognized substances protecting against oxidative and glycation modifications. Verapamil may be particularly helpful in patients with cardiovascular disease and concomitant diabetes. Studies in animal models and humans are needed to confirm verapamil’s antiglycative/antidiabetic activity.

## 1 Introduction

**FIGURE 1:**
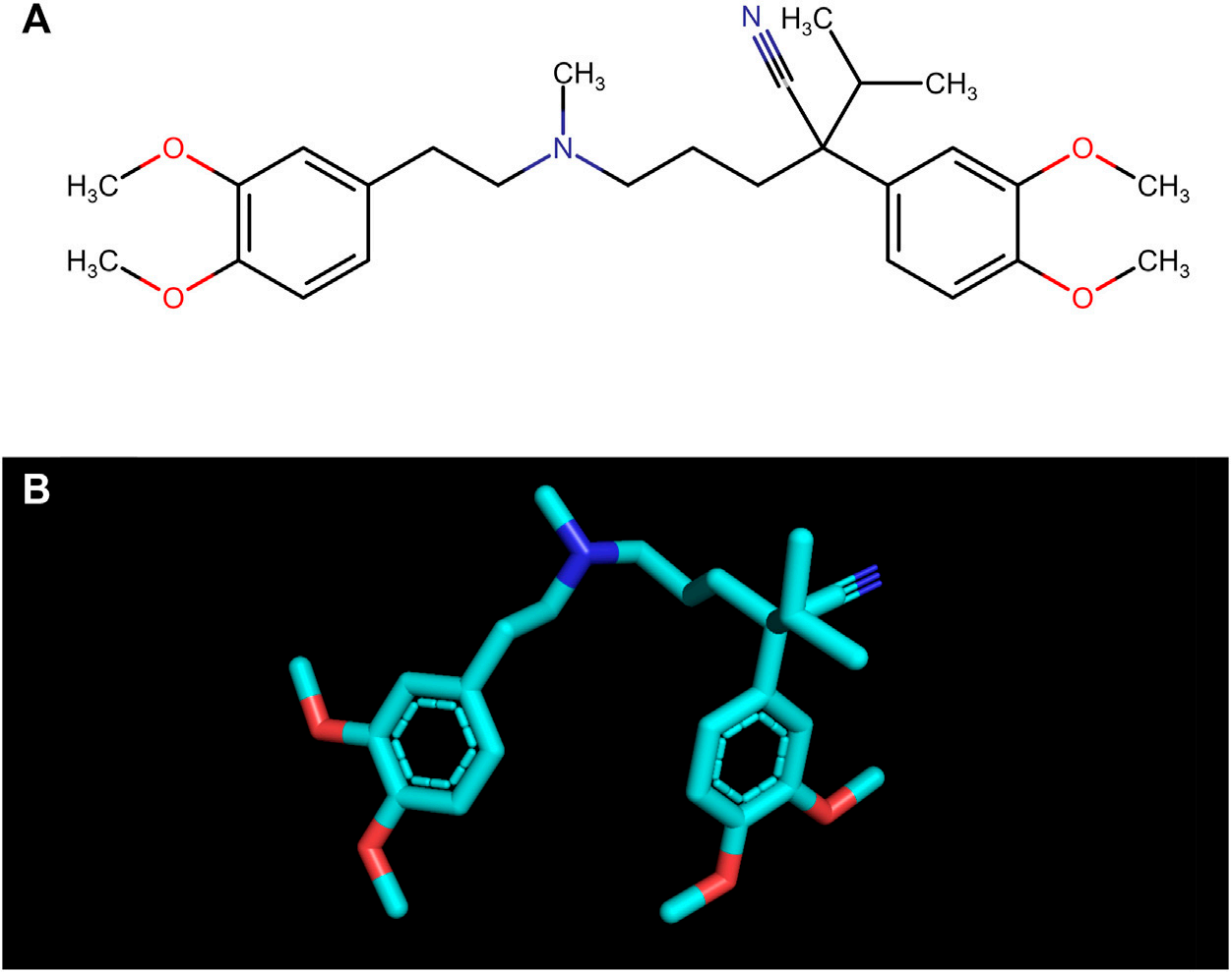
The structural formula **(A)** and the spatial structure **(B)** of verapamil ((*RS*)-2-(3,4-dimethoxyphenyl)-5-{[2-(3,4-dimethoxyphenyl)ethyl](methyl)amino}-2-(propan-2-yl)pentanenitrile; C_27_H_38_N_2_O_4_).

Verapamil ((*RS*)-2-(3,4-dimethoxyphenyl)-5-{[2-(3,4-dimethoxyphenyl)ethyl](methyl)amino}-2-(propan-2-yl)pentanenitrile; C_27_H_38_N_2_O_4_; Figure 1) is a phenylalkylamine derivative belonging to selective calcium channel blockers. The drug counteracts the increase in intracellular calcium (Ca^2+^) concentrations in the muscle cells and the cardiac stimulus-conduction system (Li and Shi, 2019). Thus, it strongly inhibits conduction in the heart, prolongs atrioventricular conduction time, and normalizes the frequency of ventricular contractions. The drug is effectively used in supraventricular arrhythmias, atrial fibrillation, and atrial flutter with rapid ventricular activity. In recommended doses, verapamil does not affect (or only slightly reduce) the regular heart rate (Guerrero and Martin, 1984; Fahie and Cassagnol, 2023). Verapamil also acts on blood vessels; nevertheless, its effect is weaker than dihydropyridine derivatives. In hypertensive patients, it reduces blood pressure with virtually no effect on those with normal blood pressure (Guerrero and Martin, 1984; Fahie and Cassagnol, 2023). The most common side effects include headache, dizziness, gastrointestinal disturbances, tachycardia, as well as a feeling of fatigue. Any side effects are relatively rare, and the benefits of taking the drug most often outweigh the potential risks (Guerrero and Martin, 1984; Fahie and Cassagnol, 2023).

**FIGURE 2:**
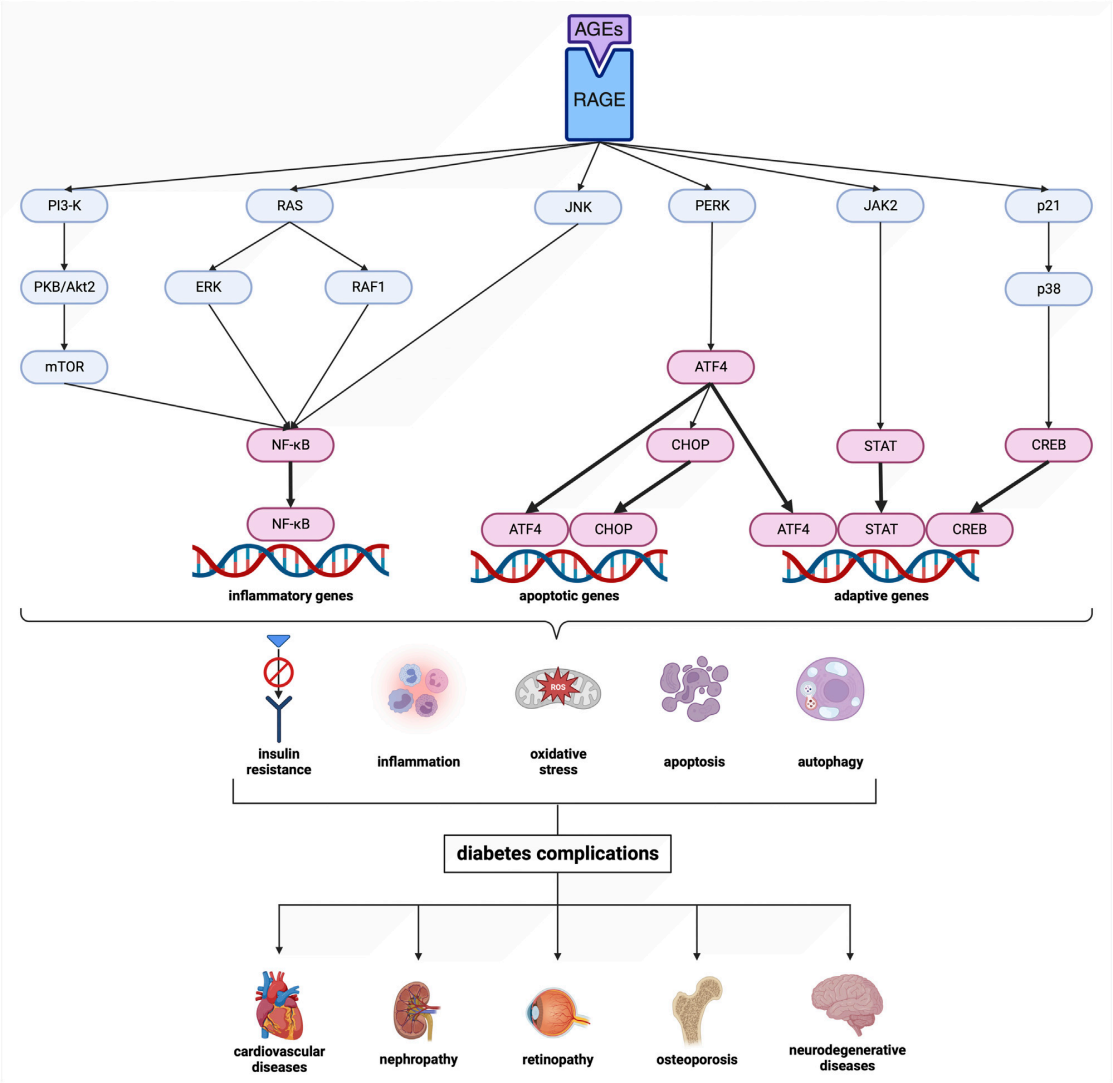
Scheme of the advanced glycation end product (AGE) pathway. Transcription factors are shown in magenta. Created with BioRender.com. AGEs, advanced glycation end products; ATF4, activating transcription factor 4; CHOP, CCAAT/enhancer binding protein homologous protein; CREB, cAMP response element-binding protein; ERK, extracellular signal-regulated kinase; JAK2, Janus kinase 2; JNK, c-Jun N-terminal kinase; mTOR, mechanistic target of rapamycin; NF-κB, nuclear factor-κB; p21, protein kinase 21; p38, protein kinase 38; PERK, protein kinase RNA-like endoplasmic reticulum kinase; PI3-K, phosphatidylinositol 3-kinase; PKB/Akt2, protein kinase B; RAF1, rapidly accelerated fibrosarcoma 1; RAGE, receptor for advanced glycation end products; RAS, RAS-related 2 protein; STAT, signal transducer and activator of transcription.

Cardiovascular diseases co-occur with disturbances in carbohydrate metabolism. Indeed, 20%–30% of patients with chronic artery disease are diagnosed with diabetes (Glovaci et al., 2019). The protein glycation/oxidation impairs the blood vessels, causing late diabetic complications. Depending on the type of damaged vessels, microangiopathies (diabetic retinopathy, diabetic kidney disease, neuropathies) and macroangiopathies (ischemic heart disease, myocardial infarction, diabetic foot syndrome, stroke) are distinguished (Ceriello and Prattichizzo, 2021). Chronic hyperglycemia leads to increased glucose flux through the polyol pathway and the formation of advanced glycation end products (AGEs) (Khalid et al., 2022). AGEs trigger the activation of pro-inflammatory pathways and enzymes, including nuclear factor-κB (NF-κB), mitogen-activated protein kinases (MAPKs), NADPH oxidase (NOX), protein kinase C (PKC), cyclooxygenase-2 (COX-2) and inducible nitric oxide synthase (iNOS). Specifically, there is a stimulation of RAGE (receptor for AGEs), which boosts the release of cytokines and adhesion molecules from immune cells (Origlia et al., 2009; Ott et al., 2014; Casella et al., 2015; Khalid et al., 2022) (Figure 2). Simultaneously, reactive oxygen species (ROS) are generated, which modify amino acids, alter prosthetic groups within proteins, and trigger fragmentation/aggregation of proteins. Advanced oxidation protein products (AOPPs) can interact with RAGE due to high structural homology to AGEs (Giacco and Brownlee, 2010; Nowotny et al., 2015). Therefore, it seems more reasonable to use the term ‘glycoxidation’ rather than glycation and oxidation separately. This mutual propulsion in diabetes and cardiovascular disease leads to chronic inflammation, endothelial dysfunction, and vascular complications (Peppa et al., 2002; Vlassara and Palace, 2003; Vlassara and Uribarri, 2004). Unfortunately, contemporary antidiabetic drugs do not have an antiglycative effect but only lower blood glucose levels. Therefore, there is an intense search for substances that could inhibit protein glycation and prevent diabetic complications.

Drug discovery is a costly and lengthy process with a high risk of failure; however, its alternative is drug repositioning, which involves finding a new therapeutic indication for marketed pharmaceuticals. Recent studies indicated the antiglycoxidant properties of cardiovascular drugs such as atorvastatin, captopril, nebivolol, probucol, or valsartan (Tanous et al., 2008; Ceron et al., 2013; Violi et al., 2014; Arjmand Abbassi et al., 2016; Mil et al., 2021, respectively). Potential antioxidant effects have also been confirmed with verapamil (Alican et al., 1994; Bukowska et al., 2008; Li et al., 2017; Jangholi et al., 2020). Chen et al. showed that verapamil improves glucose homeostasis and preserves β-cell function as monotherapy/combined with acarbose or metformin (Chen et al., 2021). However, it is unclear whether the antidiabetic activity of verapamil is due to the drug’s effect on intracellular signaling or direct inhibition of protein glycation. Given the increased incidence of type 2 diabetes, the frequent cardiovascular complications in diabetics, and the lack of an effective antidiabetic drug, assessing the antiglycoxidant effect of verapamil is fully justified. Therefore, we are the first to comprehensively evaluate the effect of verapamil on protein glycoxidation using various *in vitro* and *in silico* models.

## 2 Materials and methods

### 2.1 Chemicals and equipment

The chemicals used in this study were all of analytical grade and were procured from Sigma-Aldrich, with their location in Numbrecht, Germany/Saint Louis, MO, United States (except for bovine serum albumin [BSA], sourced from Fisher BioReagents [Pittsburgh, PA, United States of America]). Before usage, all solutions underwent sterilization by filtration through 0.2 mm membrane filters (Biosens, Warsaw, Poland). In order to distinguish the outcomes obtained for verapamil, aminoguanidine, a recognized inhibitor of protein glycation, and 6-hydroxy-2,5,7,8-tetramethylchroman-2-carboxylic acid (Trolox), an antioxidant, were used. The concentration of all additives was 1 mM, based on *in vitro* kinetic studies, and was adjusted proportionally to the high concentrations of the glycating/oxidizing agents used. The assessment of absorbance and fluorescence was carried out utilizing an M200 PRO multimode microplate reader manufactured by Tecan Group Ltd., located in Männedorf, Switzerland.

### 2.2 BSA model

Following a previously documented procedure, bovine serum albumin (BSA) glycation and oxidation were conducted (Sadowska-Bartosz et al., 2014a; Sadowska-Bartosz et al., 2014b; Pawlukianiec et al., 2020; Mil et al., 2021; Nesterowicz et al., 2023a; Nesterowicz et al., 2023b). Initially, BSA with a purity of over 98%, devoid of proteases and fatty acids, and at a concentration of 90 μmol/L, was dissolved in a sodium phosphate buffer (0.1 M, pH 7.4) containing sodium azide (1 mM) as a preservative. As glycating factors, sugars (glucose [Glu], fructose [Fru], and ribose [Rib] at a concentration of 0.5 M), as well as aldehyde (glyoxal [GO] at a concentration of 2.5 mM), were used. Meanwhile, chloramine T (ChT) at a concentration of 20 mM was used as an oxidizing agent. The incubation was carried out in sealed vials under dark conditions with continuous shaking (50 rpm, 37°C) for 6 days with sugars, 12 hours with GO, and 1 hour with ChT (Sadowska-Bartosz et al., 2014a; Sadowska-Bartosz et al., 2014b; Pawlukianiec et al., 2020; Mil et al., 2021; Nesterowicz et al., 2023a; Nesterowicz et al., 2023b).

The concentrations of glycating/oxidizing factors used were much higher than their physiological levels. Nevertheless, such conditions effectively simulate the physiological processes in the body over several months in a relatively short period (Biedrzycki et al., 2023). Such conditions are routinely used to assess the antiglycoxidant properties of novel substances. All additives were used at a concentration of 1 mM, based on *in vitro* kinetic studies, and were proportionally adjusted to match the high levels of the glycating agents (Sadowska-Bartosz et al., 2014a; 2014b; Pawlukianiec et al., 2020; Mil et al., 2021; Nesterowicz et al., 2023a; 2023b). The entire study was conducted in three independent experiments, each repeated twice.

### 2.3 Oxidation products

The concentration of total thiols (TTs) was analyzed using a spectrophotometer at 412 nm using Ellman’s reagent. The level of TTs was quantified based on the standard curve of reduced glutathione (GSH) (Ellman, 1959).

For the assessment of protein carbonyls (PCs) concentration, the reaction between carbonyls in oxidatively damaged proteins and 2,4-dinitrophenylhydrazine (DNPH) was used. The absorbance of the reaction product was measured colorimetrically at a wavelength of 355 nm. The absorption coefficient for 2,4-DNPH (22,000 M^-1^cm^-1^) served as the standard (Reznick and Packer, 1994).

A spectrophotometric assay was performed to investigate the level of advanced oxidation protein products (AOPPs). The investigated samples were diluted with phosphate-buffered saline (PBS) in a 1:5 ratio (v/v), along with standard solutions (ranging from 0 to 100 μmol/L), and placed in a 96-well microplate. Subsequently, 10 µL of 1.16 M potassium iodide and 20 µL of acetic acid were added to the wells. The absorbance at 340 nm wavelength was immediately determined using a microplate reader, compared to the blank solution (200 µL PBS, 10 µL potassium iodide, 20 µL acetic acid). The ChT solutions exhibited linear absorbance from 0 to 100 μmol/L (Skrha et al., 2007; Maciejczyk et al., 2022).

### 2.4 Glycoxidation products

Fluorescence emission and excitation were utilized to evaluate four compounds: tryptophan (TRY), kynurenine (KN), N-formylkynurenine (NFK), and dityrosine (DT). The measurements were performed at specific wavelength pairs: 95 nm excitation and 340 nm emission for TRY, 365 nm excitation and 480 nm emission for KN, 325 nm excitation and 434 nm emission for NFK, and 330 nm excitation and 415 nm emission for DT. Before the measurements, the analyzed solutions were diluted with 0.1 M sulphuric acid (H_2_SO_4_) at a ratio of 1:5 (v/v). The results were then standardized using the fluorescence of 0.1 mg/mL quinine sulfate in 0.1 M H_2_SO_4_ as a reference (Hawkins et al., 2009).

### 2.5 Glycation products

The total quantity of Amadori products (APs) was evaluated through a colorimetric nitroblue tetrazolium (NBT) assay. The absorbance was measured at a wavelength of 525 nm, using the monoformazan extinction coefficient (12,640 M^-1^ cm^-1^) for estimation (Sharma et al., 2002).

To detect fluorescence emitted during the binding of β-amyloid (βA) fibrils/oligomers, thioflavin T was examined. A mixture of thioflavin T (10 µL) and samples (90 µL) was prepared and placed on a microplate. The fluorescence intensity was quantified at a wavelength of 385/485 nm (Levine, 1993; Hudson et al., 2009).

The content of advanced glycation end products (AGEs) was investigated using a spectrofluorometer. Specific for AGEs fluorescence was analyzed at a wavelength of 440/370 nm. Before the study, the analyzed samples were diluted with PBS in a 1:5 (v/v) ratio (Münch et al., 1997; Skrha et al., 2007).

### 2.6 Molecular docking

Molecular docking is a computational technique that predicts the favorable binding position of a ligand to a macromolecule. As the receptors to study their interactions with verapamil, BSA, glycoside hydrolases: α-amylase (αA; EC 3.2.1.1), α-glucosidase (αG; EC 3.2.1.20), and sucrase-isomaltase (IS; EC 3.2.1.10), as well as seventeen AGE pathway proteins: receptor for advanced glycation end products (RAGE), signal transducer and activator of transcription (STAT), Janus kinase 2 (JAK2), cAMP response element-binding protein (CREB), activating transcription factor 4 (ATF4), protein kinase RNA-like endoplasmic reticulum kinase (PERK), protein kinase 38 (p38), extracellular signal-regulated kinase (ERK), phosphatidylinositol 3-kinase (PI3-K), protein kinase B (PKB/Akt2), c-Jun N-terminal kinase (JNK), CCAAT/enhancer binding protein homologous protein (CHOP), nuclear factor-κB (NF-κB), protein kinase 21 (p21), rapidly accelerated fibrosarcoma 1 (RAF1), RAS-related 2 protein (RAS), mechanistic target of rapamycin (mTOR) were used in our study.

The 3D structures of the receptors were retrieved from the Protein Data Bank (PDB) in. pdb formats (Table 1–3). The 3D structure of verapamil (PubChem CID: 2520) was obtained from the National Library of Medicine (NLM) website as a. sdf file. Preprocessing of the proteins involved the removal of all water molecules, polar hydrogens, and Kollman charges using the AutoDock MGL Tools. The prepared proteins were saved in. pdbqt format. During the molecular docking simulations, we set the exhaustiveness parameter to a value of 8 in both receptors. AutoDock Vina was used, and the grid size was set at 40 × 40 × 40. We utilized the PyMOL 2.5 program to visualize the molecular docking results. This allowed us to examine the binding interactions between proteins and verapamil at the molecular level (Oso and Olaoye, 2020; Sindhuja et al., 2021; Nesterowicz et al., 2023a; Nesterowicz et al., 2023b).

**TABLE 1:** Results of molecular docking simulations between verapamil and bovine serum albumin (BSA). LYS, lysine.

Name of protein	RCSB ID	Affinity (kcal/mol)	Number of polar contacts	Amino acid residues
bovine serum albumin (BSA)	4F5S	−7.6	1	LYS-136

**TABLE 2:** Results of molecular docking simulations between verapamil and glycosidases. ASN, asparagine; HIS, histidine; LYS, lysine.

Enzyme number	Name of enzyme (EC number)	RCSB ID	Affinity (kcal/mol)	Number of polar contacts	Amino acid residues
1	α-amylase (αA; EC 3.2.1.1)	1HNY	−7.0	1	HIS-201
2	α-glucosidase (αG; EC 3.2.1.20)	5KZW	−6.3	4	GLY-651, 3xSER-676
3	sucrase-isomaltase (IS; EC 3.2.1.10)	3LPO	−6.0	2	2xASN-43

**TABLE 3:** Results of molecular docking simulations between verapamil and advanced glycation end product (AGE) pathway proteins. ARG, arginine; ASN, asparagine; ASP, aspartic acid; ATF4, activating transcription factor 4; CHOP, CCAAT/enhancer binding protein homologous protein; CREB, cAMP response element-binding protein; DA, deoxyadenosine; ERK, extracellular signal-regulated kinase; GLN, glutamine; GLU, glutamic acid; JAK2, Janus kinase 2; JNK, c-Jun N-terminal kinase; LYS, lysine; mTOR, mechanistic target of rapamycin; NF-κB, nuclear factor-κB; p21, protein kinase 21; p38, protein kinase 38; PERK, protein kinase RNA-like endoplasmic reticulum kinase; PI3-K, phosphatidylinositol 3-kinase; PKB/Akt2, protein kinase B; RAF1, rapidly accelerated fibrosarcoma 1; RAGE, receptor for advanced glycation end products; RAS, RAS-related 2 protein; SER, serine; STAT, signal transducer and activator of transcription.

Number of protein	Name of protein	RCSB ID	Affinity (kcal/mol)	Number of polar contacts	Residues
1	RAGE	3O3U	−6.2	1	ASN-150
2	STAT	3WWT	−5.2	1	SER-2
3	JAK2	6VN8	−7.6	0	
4	CREB	4NYX	−5.0	0	
5	ATF4	1CI6	−4.6	0	
6	PERK	4 × 7K	−5.5	3	ASN-937, 2xLYS-939
7	p38	5OMG	−6.2	2	2xGLN-202
8	ERK	5LCJ	−6.0	2	GLU-33, ARG-191
9	PI3-K	6BTY	−5.7	3	3xARG-1610
10	PKB/Akt2	2UZR	−5.7	3	3xARG-25
11	JNK	4W4V	−6.6	1	SER-96
12	CHOP	3T92	−6.7	1	ASP-7
13	NF-κB	1A3Q	−7.3	3	SER-220, SER-226, DA-509
14	p21	821P	−5.1	0	
15	RAF1	1C1Y	−5.4	4	2xGLN-25, GLN-43, GLN-66
16	RAS	4L8G	−5.3	3	ASP-153, ASP-154, ARG-161
17	mTOR	5WBH	−6.6	0	

### 2.7 Statistics

The statistical analysis used GraphPad Prism 8.3.0 (GraphPad Software, La Jolla, CA, United States of America). The results were presented as percentages relative to their respective control values (BSA + glycating/oxidizing factor [Glc, Fru, Rib, GO, and ChT]). The distribution of results was assessed using the Shapiro-Wilk test. Additionally, we examined the homogeneity of variance through Levine’s test. A one-way analysis of variance (ANOVA) was conducted to compare the differences between groups, followed by Tukey’s *post hoc* test for multiple comparisons. Statistical significance was set at *p* < 0.05, and we also used the multiplicity-adjusted *p*-value.

## 3 Results

### 3.1 BSA model

Of all cellular biomolecules, proteins are particularly susceptible to oxidation and glycation. BSA is a commonly used model protein in *in vitro* studies due to its stability, high structural homology to human albumin, and easy availability. Our experiment treated BSA in various ways to assess verapamil’s impact on protein glycoxidation rate. Three sugars—glucose (Glu), fructose (Fru), ribose (Rib), as well as aldehyde—glyoxal (GO), were used as glycating agents. Chloramine T (ChT) was used as an oxidizing agent. BSA was treated differently because the specific glycation/oxidation factors have varying biological reactivity towards albumin (Sadowska-Bartosz et al., 2014a; Sadowska-Bartosz et al., 2014b; Jahan and Choudhary, 2015).

The biomarkers we evaluated can be divided into oxidation-specific (TTs, PCs, AOPPs), glycoxidation-specific (TRY, KN, NFK, DT), as well as glycation-specific (APs, βA, AGEs). Oxidative stress causes free thiol groups (TTs) to oxidize or combine via disulfide bridges with other amino acids. Arginine (ARG), lysine (LYS), proline (PRO), and threonine (THR) undergo carbonylation with the formation of PCs. However, the final products of protein oxidation are AOPPs (Stadtman and Berlett, 1998; Bader and Grune, 2006). On the other hand, carbonyl stress induces protein glycation through the Maillard reaction. The early stages produce Schiff bases, which then regroup into APs. Prolonged exposure of proteins to glycating agents causes α-helix transition to a linear structure, giving rise to the formation of βA. The late stage involves oxidation, polymerization, dehydration, and condensation reactions with other amino groups. This results in the formation of the final glycation products—AGEs (Shen et al., 2020; Shin et al., 2023). In glycoxidation processes, the kynurenine pathway converts TRY via the intractable NFK to KN. Meanwhile, two tyrosine (TYR) residues form a cross-link in the form of a dityrosine bridge (DT) (Stadtman and Levine, 2003; Poojary and Lund, 2022).

#### 3.1.1 Glucose (Glc)-induced glycation

Of all circulating sugars, D-glucose is found in the human body in the highest concentration (Mou et al., 2022). Physiologically, fasting blood glucose level is 3.9–5.6 mmol/; however, only the linearized form of glucose has a free aldose group. Thus, it is not surprising that protein glycation *in vivo* occurs slowly. It increases significantly under hyperglycemic conditions (McMillin, 1990; Chen et al., 2019; Reddy et al., 2022).

**FIGURE 3:**
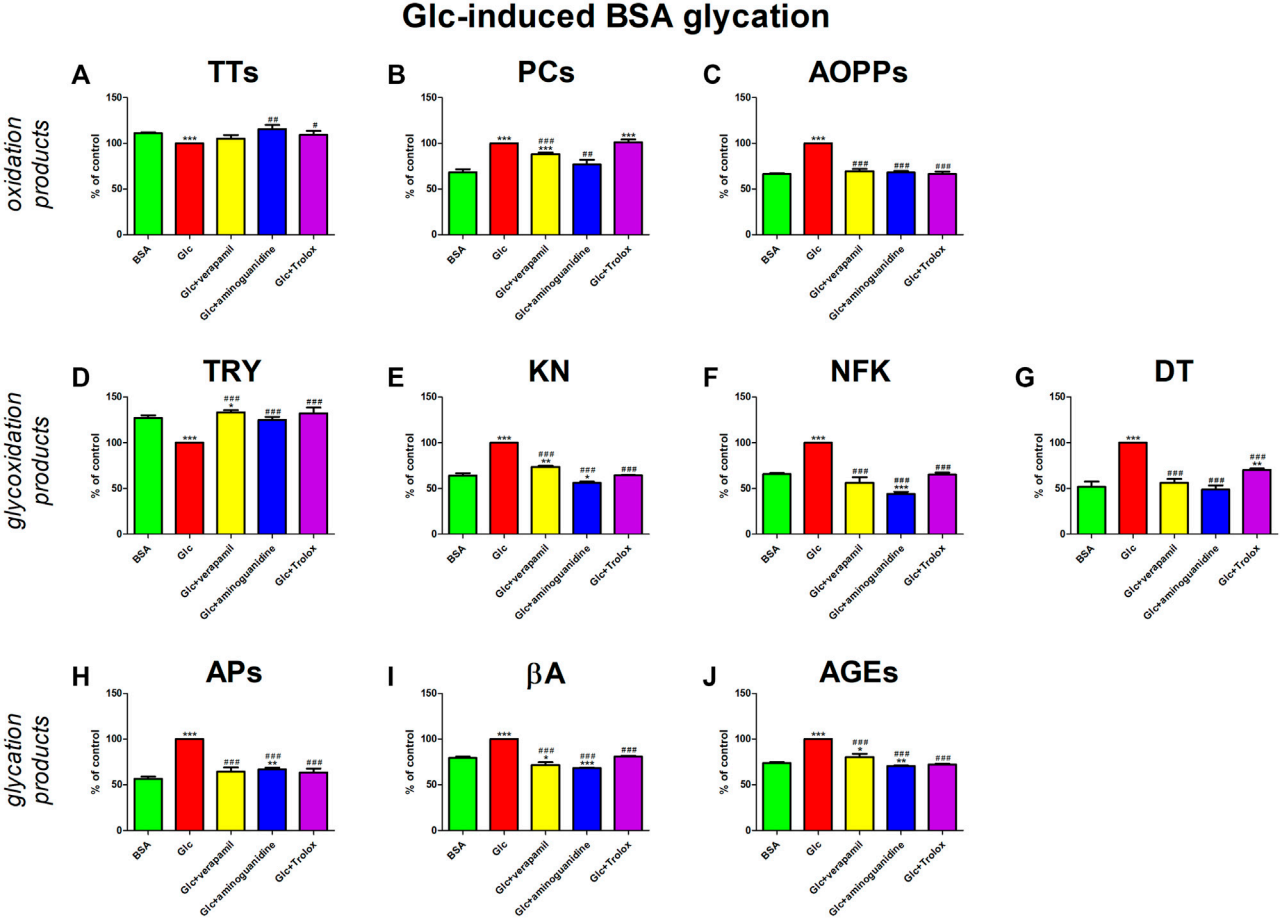
Verapamil’s impact on protein oxidation **(A–C)**, glycoxidation **(D–G)**, and glycation **(H–J)** in glucose (Glc)-glycated bovine serum albumin (BSA). AGEs, advanced glycation end products; AOPPs, advanced oxidation protein products; APs, Amadori products; βA, β-amyloid; BSA, bovine serum albumin; DT, dityrosine; Glc, glucose; KN, kynurenine; NFK, N-formylkynurenine; PCs, protein carbonyls; Trolox, 6-hydroxy-2,5,7,8-tetramethylchroman-2-carboxylic acid; TRY, tryptophan; TTs, total thiols. *p < 0.05 vs. negative control (BSA); **p < 0.01 vs. negative control (BSA); ***p < 0.001 vs. negative control (BSA); #*p* < 0.05 vs. positive control (BSA + Glc); ##*p* < 0.01 vs. positive control (BSA + Glc); ###*p* < 0.001 vs. positive control (BSA + Glc).

The addition of Glc to the BSA significantly enhanced protein oxidation (↓TTs, ↑PCs, ↑AOPPs), glycoxidation (↓TRY, ↑KN, ↑NFK, ↑DT), and glycation (↑APs, ↑βA, ↑AGEs). Verapamil counteracted these changes—efficiently increased TRY and meaningfully decreased βA against both Glc + BSA and BSA. The studied drug markedly reduced AOPPs, NFK, DT, and APs relative to Glc + BSA, to values not substantially different from BSA alone. Verapamil significantly reduced PCs, KNs, and AGEs relative to Glc + BSA, but values remained relevantly higher than BSA (Figure 3).

Verapamil’s activity was also compared to the model substances (aminoguinidine and Trolox). Aminoguanidine neutralizes α,β-dicarbonyls competing with glycating factors, while Trolox scavenges ROS and chelates redox-active metal ions (Reszka et al., 1999; Hamad et al., 2010; Bavkar et al., 2019). In our study, the glycoxidation inhibition rate by verapamil is comparable to the effects of aminoguanidine and Trolox.

#### 3.1.2 Fructose (Fru)-induced glycation

Fru is a ketohexose found in the blood of a healthy person at concentrations of up to 100 μM, which is about 50 times lower than glucose. However, *in vitro* studies have shown that fructose acts several times more saturated compared to glucose. In people with diabetes, fructose is produced via the polyol pathway. Consequently, in some tissues, its concentration reaches the same magnitude as glucose (Sadowska-Bartosz et al., 2014a; Mou et al., 2022).

**FIGURE 4:**
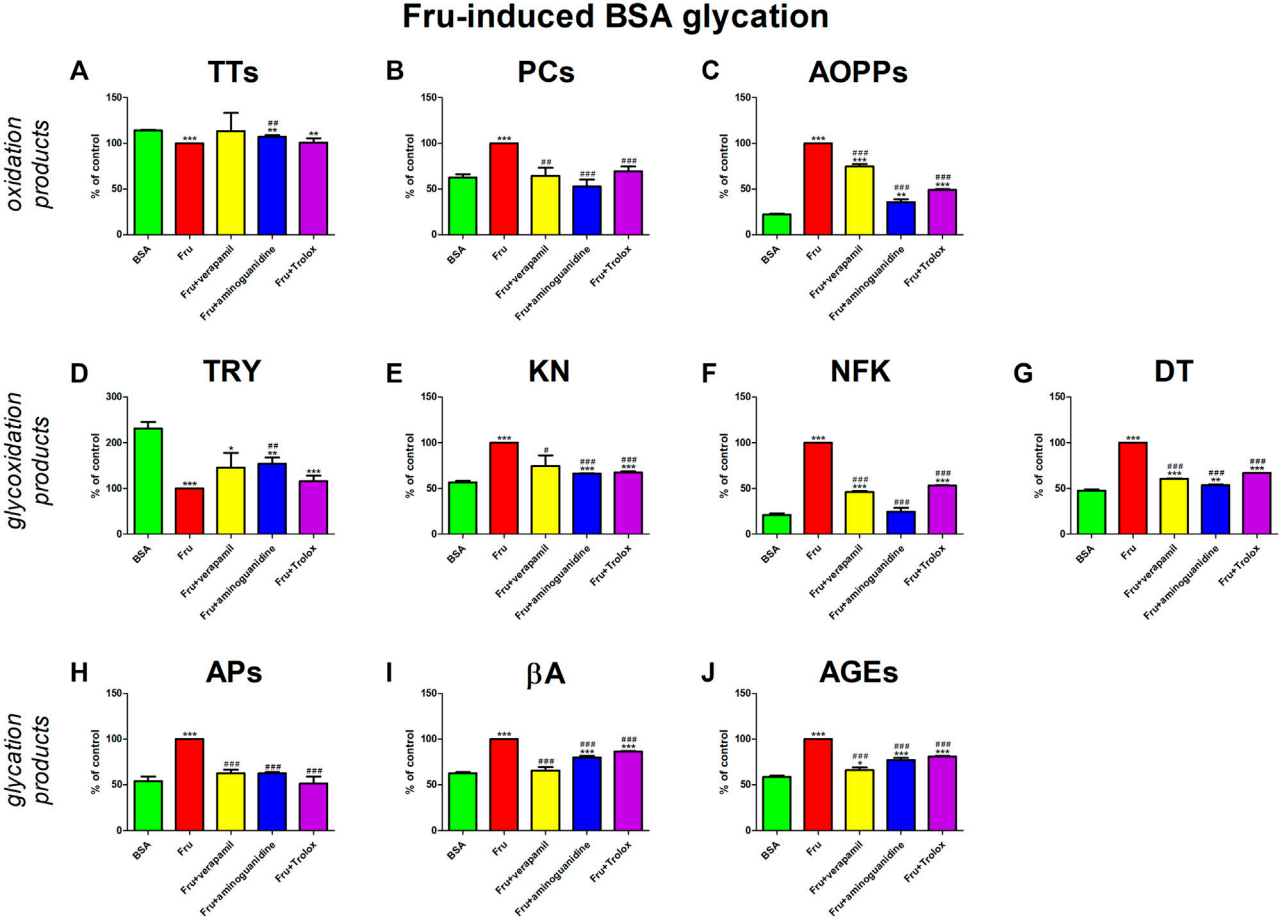
Verapamil’s impact on protein oxidation **(A–C)**, glycoxidation **(D–G)**, and glycation **(H–J)** in fructose (Fru)-glycated bovine serum albumin (BSA). AGEs, advanced glycation end products; AOPPs, advanced oxidation protein products; APs, Amadori products; βA, β-amyloid; BSA, bovine serum albumin; DT, dityrosine; Fru, fructose; KN, kynurenine; NFK, N-formylkynurenine; PCs, protein carbonyls; Trolox, 6-hydroxy-2,5,7,8-tetramethylchroman-2-carboxylic acid; TRY, tryptophan; TTs, total thiols. *p < 0.05 vs. negative control (BSA); **p < 0.01 vs. negative control (BSA); ***p < 0.001 vs. negative control (BSA); #*p* < 0.05 vs. positive control (BSA + Glc); ##*p* < 0.01 vs. positive control (BSA + Glc); ###*p* < 0.001 vs. positive control (BSA + Glc).

In our study, Fru significantly enhances parameters of oxidative stress (↓TTs, ↑PCs, ↑AOPPs) and carbonyl stress (↓TRY, ↑KN, ↑NFK, ↑DT, APs, ↑βA, ↑AGEs). Verapamil and model substances (aminoguanidine and Trolox) inhibited the above processes. They relevantly diminished PCs, KN, APs, and βA compared to Fru + BSA, to a level that did not significantly vary from BSA. Verapamil effectively attenuated AOPPs, NFK, DT, KNs, as well as AGEs versus Fru + BSA, but not in comparison with BSA alone (Figure 4).

#### 3.1.3 Ribose (Rib)-induced glycation

Rib, a five-carbon aldose, is found in human blood in similar concentrations to fructose. However, *in vitro* studies in a BSA model have shown that ribose induces the glycation of more LYS of BSA and causes more AGE formation than fructose. Unlike hexoses (glucose and fructose), ribose also tended to convert BSA to amyloid-like monomers (Wei et al., 2009; Sadowska-Bartosz et al., 2014a; Mou et al., 2022).

**FIGURE 5:**
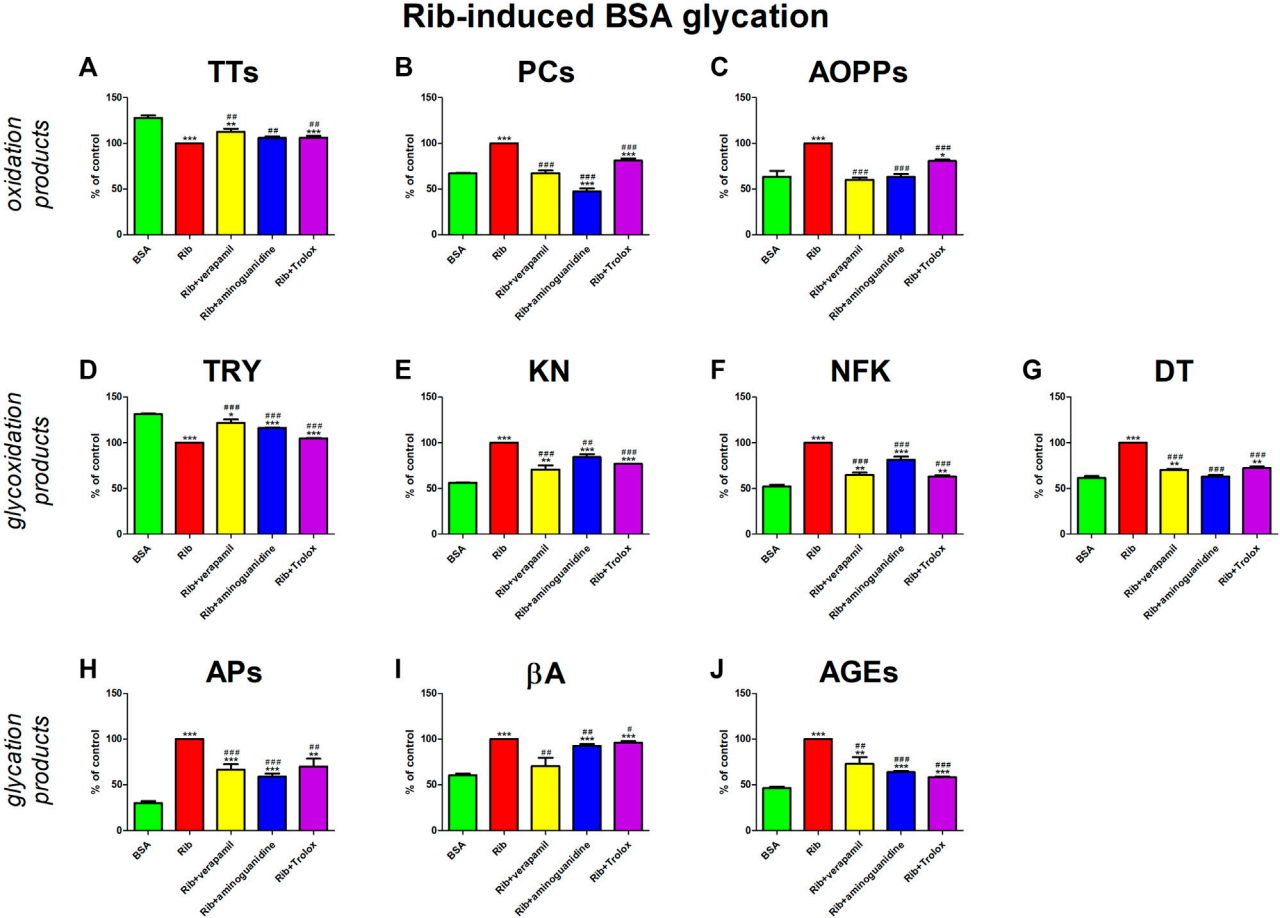
Verapamil’s impact on protein oxidation **(A–C)**, glycoxidation **(D–G)**, and glycation **(H–J)** in ribose (Rib)-glycated bovine serum albumin (BSA). AGEs, advanced glycation end products; AOPPs, advanced oxidation protein products; APs, Amadori products; βA, β-amyloid; BSA, bovine serum albumin; DT, dityrosine; KN, kynurenine; NFK, N-formylkynurenine; PCs, protein carbonyls; Rib, ribose; Trolox, 6-hydroxy-2,5,7,8-tetramethylchroman-2-carboxylic acid; TRY, tryptophan; TTs, total thiols. *p < 0.05 vs. negative control (BSA); **p < 0.01 vs. negative control (BSA); ***p < 0.001 vs. negative control (BSA); #*p* < 0.05 vs. positive control (BSA + Glc); ##*p* < 0.01 vs. positive control (BSA + Glc); ###*p* < 0.001 vs. positive control (BSA + Glc).

The addition of Rib to the BSA markedly elevated protein oxidation (↓TTs, ↑PCs, ↑AOPPs), glycoxidation (↓TRY, ↑KN, ↑NFK, ↑DT), and also glycation (↑APs, ↑βA, ↑AGEs) products. All additives (verapamil, aminoguanidine, Trolox) mitigate the alterations. Verapamil substantially reduced PCs, AOPPs, and βA against Rib + BSA, rendering these parameters statistically equal to the values of BSA alone. The drug significantly increased TTs and TRY, as well as decreased all kinurenine pathway metabolites, APs, and also AGEs when compared to Rib + BSA. However, the markers were still meaningfully diverse from BSA (Figure 5).

#### 3.1.4 Glyoxal (GO)-induced glycation

GO is an endogenous α-oxoaldehyde, a highly reactive dicarbonyl compound formed by the degradation of early Maillard reaction products. In addition to protein glycation, dicarbonyls are also produced in the glycolytic pathway, the pentose cycle, the sorbitol cycle, and the catabolism of ketone bodies. Dicarbonyl compounds are direct precursors of AGEs (Voziyan et al., 2014; Alouffi and Khan, 2020). In healthy individuals, AGEs are degraded and eliminated from the body physiologically, and they do not suffer the harmful effects of protein glycation. However, in the presence of chronic diseases and during aging, the glycation process intensifies, and AGE elimination systems are weakened (Peppa and Mavroeidi, 2021; Reddy et al., 2022).

**FIGURE 6:**
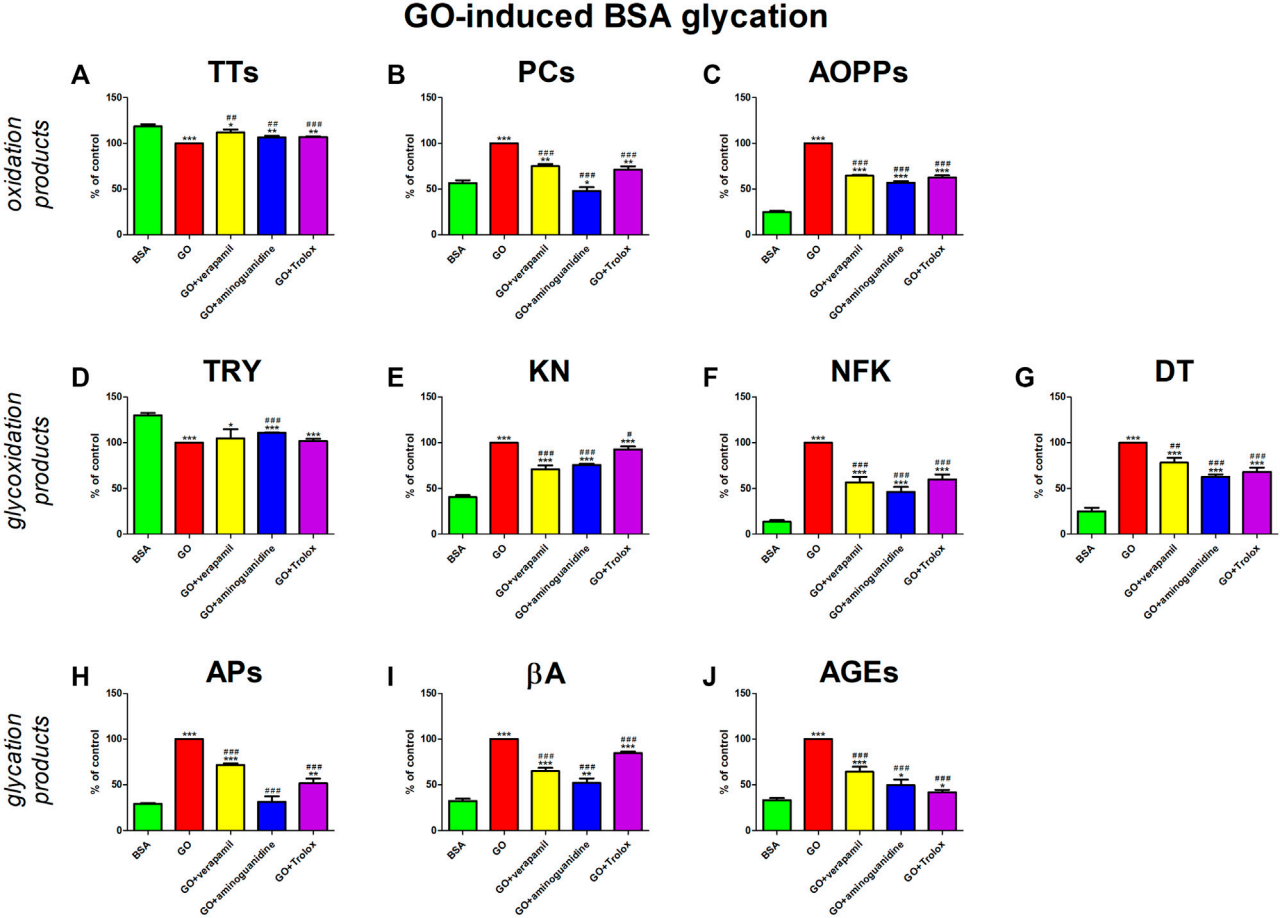
Verapamil’s impact on protein oxidation **(A–C)**, glycoxidation **(D–G)**, and glycation **(H–J)** in glyoxal (GO)-glycated bovine serum albumin (BSA). AGEs, advanced glycation end products; AOPPs, advanced oxidation protein products; APs, Amadori products; βA, β-amyloid; BSA, bovine serum albumin; DT, dityrosine; GO, glyoxal; KN, kynurenine; NFK, N-formylkynurenine; PCs, protein carbonyls; Trolox, 6-hydroxy-2,5,7,8-tetramethylchroman-2-carboxylic acid; TRY, tryptophan; TTs, total thiols. *p < 0.05 vs. negative control (BSA); **p < 0.01 vs. negative control (BSA); ***p < 0.001 vs. negative control (BSA); #*p* < 0.05 vs. positive control (BSA + Glc); ##*p* < 0.01 vs. positive control (BSA + Glc); ###*p* < 0.001 vs. positive control (BSA + Glc).

GO treatment occasioned a signal rise in oxidative stress (↓TTs, ↑PCs, ↑AOPPs) and carbonyl stress (↓TRY, ↑KN, ↑NFK, ↑DT, ↑APs, ↑βA, ↑AGEs). The studied drug suppressed these conversions. Verapamil significantly counteracted all changes except for TRY induced by GO. Nevertheless, none of the parameters reach the value in BSA alone. The effect of verapamil is comparable to substances with proven antiglycative (aminoguanidine) and antioxidant activity (Trolox) (Figure 6).

#### 3.1.5 Chloramine T (ChT)-induced oxidation

ChT is a synthetic, powerful oxidant. It is the source of the main physiological active chlorine species (ACS): hypochlorous acid (HOCl) and chloramine (NH_2_Cl) (Dannan et al., 1992; Tongul et al., 2018).

**FIGURE 7:**
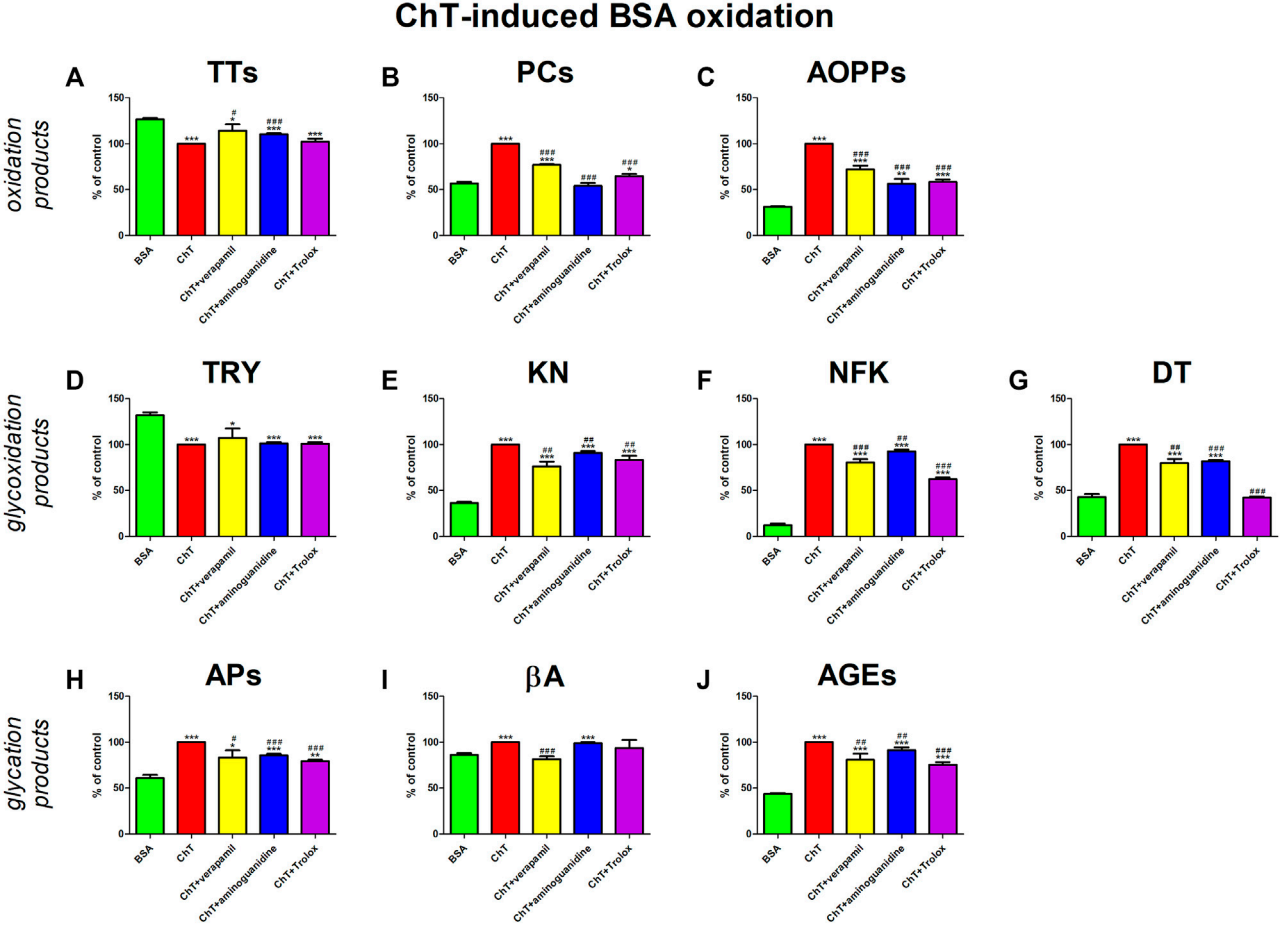
Verapamil’s impact on protein oxidation **(A–C)**, glycoxidation **(D–G)**, and glycation **(H–J)** in chloramine T (ChT)-oxidated bovine serum albumin (BSA). AGEs, advanced glycation end products; AOPPs, advanced oxidation protein products; APs, Amadori products; βA, β-amyloid; BSA, bovine serum albumin; ChT, chloramine T; DT, dityrosine; KN, kynurenine; NFK, N-formylkynurenine; PCs, protein carbonyls; Trolox, 6-hydroxy-2,5,7,8-tetramethylchroman-2-carboxylic acid; TRY, tryptophan; TTs, total thiols. *p < 0.05 vs. negative control (BSA); **p < 0.01 vs. negative control (BSA); ***p < 0.001 vs. negative control (BSA); #*p* < 0.05 vs. positive control (BSA + Glc); ##*p* < 0.01 vs. positive control (BSA + Glc); ###*p* < 0.001 vs. positive control (BSA + Glc).

The addition of ChT to the BSA significantly enhanced oxidation (↓TTs, ↑PCs, ↑AOPPs), glycoxidation (↓TRY, ↑KN, ↑NFK, ↑DT), and glycation (↑APs, ↑βA, ↑AGEs) processes. The presence of verapamil counteracted these changes. Verapamil efficiently reduced βA relative to ChT + BSA to values not substantially different from BSA alone. The tested drug markedly potentiated TTs and also suppressed PCs, AOPPs, KN, NFK, DT, APs, and AGEs besides ChT + BSA, but the markers were markedly various than BSA. The effect of verapamil is comparable to aminoguanidine and Trolox (Figure 7).

### 3.2 Molecular docking analysis

**FIGURE 8:**
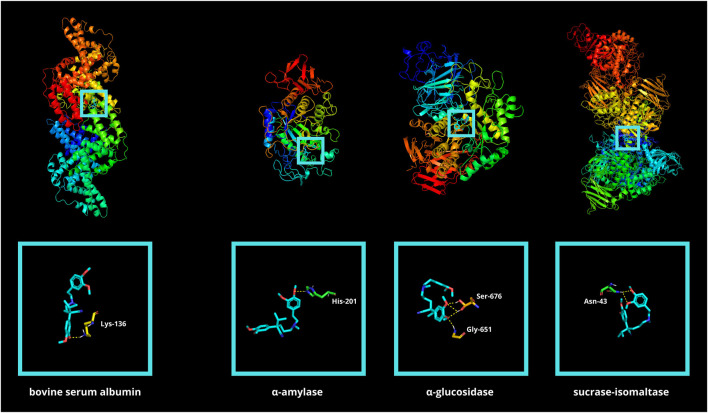
Visualization of verapamil docking sites (modes 1) in bovine serum albumin (BSA) as well as in glycosidases: α-amylase (αA), α-glucosidase (αG), and sucrase-isomaltase (SI). The spatial structure of verapamil has been marked in cyan color. ASN, asparagine; HIS, histidine; LYS, lysine.

First, molecular docking simulation was performed between verapamil and BSA (Table 1; Figure 8). The goal was to assess the affinity of verapamil for the binding sites of the albumin. Competition by binding sites could potentially protect the protein from the attachment of glycating/oxidizing agents (Bavkar et al., 2019). The simulation showed that verapamil bound preferentially to amino acids particularly prone to glycoxidative damage (LYS, TYR, ARG, phenylalanine [PHE]) (Stadtman and Levine, 2003). *In silico* analysis demonstrated the strongest drug’s binding affinity to a BSA particle at a score of −7.6 kcal/mol. Among the various docking sites analyzed, only this site exhibited root-mean-square deviations of atomic positions (RMSD) below 3. The site displayed a single polar contact with the BSA particle, involving just LYS, at position 136 (Table 1; Supplementary Table S1; Figure 8).

Molecular docking between verapamil and selected glycosidases was also conducted. α-amylase (αA), α-glucosidase (αG), as well as sucrase-isomaltase (SI), are digestive enzymes responsible for breaking down polysaccharides. Disruption of enzyme function results in reduced conversion of complex sugars to easily digestible simple saccharides. Verapamil showed low binding energy for all the hydrolases above (−7.0, −6.3, and −6.0 kcal/mol, respectively) (Table 2; Figure 8). It is well known that if the energy of the ligand-receptor complex is decreasing, there is better docking and higher affinity. The good affinity of verapamil to the enzymes indicates the potential for inhibiting their activity. Thus, the potential hypoglycemic effect of verapamil *in vivo* may be related to its antiglycative properties. The greatest biological significance in the digestion of carbohydrates is played by αG. The first poze (with the highest affinity) had an RMSD of less than 3. However, this αG particle’s docking site had as many as four polar interactions with verapamil through glycine (GLY)-651 and serine (SER)-676 (three contacts) (Hossain et al., 2020; Oso and Olaoye, 2020) (Table 2; Supplementary Table S2; Figure 8).

**FIGURE 9:**
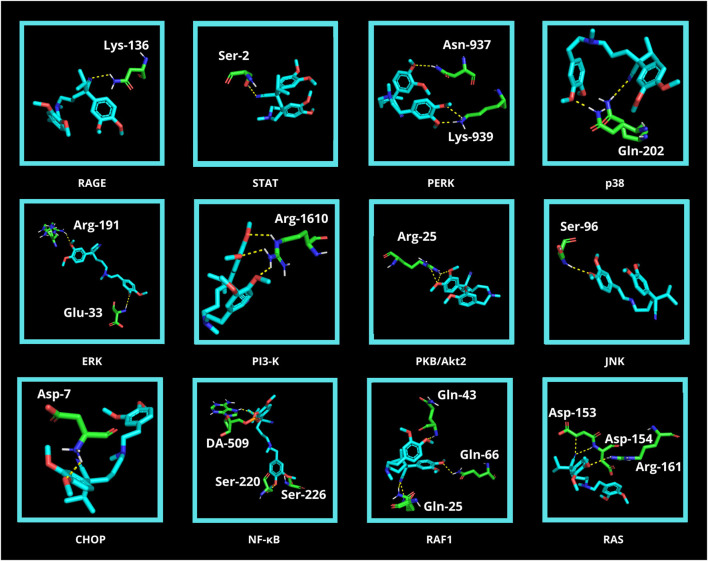
Visualization of verapamil docking sites (modes 1) in advanced glycation end product (AGE) pathway proteins. The spatial structure of verapamil has been marked in cyan color. Janus kinase 2 (JAK2), cAMP response element-binding protein (CREB), activating transcription factor 4 (ATF4), protein kinase 21 (p21), and mechanistic target of rapamycin (mTOR) did not show any polar contact. ARG, arginine; ASN, asparagine; ASP, aspartic acid; CHOP, CCAAT/enhancer binding protein homologous protein; DA, deoxyadenosine; ERK, extracellular signal-regulated kinase; GLN, glutamine; GLU, glutamic acid; JNK, c-Jun N-terminal kinase; LYS, lysine; NF-κB, nuclear factor-κB; p38, protein kinase 38; PERK, protein kinase RNA-like endoplasmic reticulum kinase; PI3-K, phosphatidylinositol 3-kinase; PKB/Akt2, protein kinase B; RAF1, rapidly accelerated fibrosarcoma 1; RAGE, receptor for advanced glycation end products; RAS, RAS-related 2 protein; SER, serine; STAT, signal transducer and activator of transcription.

Finally, *in silico* analysis was conducted for AGE pathway proteins (Table 3; Figure 9). The receptor for advanced glycation end products (RAGE) is a central hub in the AGE signaling cascade, initiating several pathways with intertwined interactions. Through activation of the phosphatidylinositol 3-kinase (PI3-K)/protein kinase B (PKB/Akt2)/mechanistic target of rapamycin (mTOR) pathway, induction of nuclear factor-κB (NF-κB) expression occurs. This effect also follows stimulation of RAS-related 2 protein (RAS) (via extracellular signal-regulated kinase [ERK] or rapidly accelerated fibrosarcoma 1 [RAF1]) as well as c-Jun N-terminal kinase (JNK). NF-κB modulates inflammatory genes. In contrast, RAGE-mediated activation of protein kinase RNA-like endoplasmic reticulum kinase (PERK) leads to overexpression of activating transcription factor 4 (ATF-4) which is both a proapoptotic and also adaptive factor. ATF-4 further stimulates the biosynthesis of another apoptosis-inducing factor, CCAAT/enhancer binding protein homologous protein (CHOP). RAGE also stimulates the Janus kinase 2 (JAK2)/signal transducer and activator of the transcription (STAT) pathway as well as increases levels of protein kinase 21 (p21). p21, via protein kinase 38 (p38), enhances CREB expression. STAT and CREB (together with the aforementioned ATF-4) modulate the activity of anti-apoptotic genes. The action of transcription factors leads to insulin resistance, inflammation, oxidative stress, apoptosis, and also autophagy. In diabetes, AGEs-RAGE signaling is overexpressed, which is a direct cause of diabetic complications (Pathomthongtaweechai and Chutipongtanate, 2020; Shen et al., 2020; Sindhuja et al., 2021) (Figure 2). Verapamil showed satisfactory affinity (no lower than −4.6 kcal/mol) for all seventeen proteins, possibly restoring their initial physiological function. The drug bound most strongly to Janus kinase 2 (JAK2; −7.6 kcal/mol; no polar contacts), as well as NF-κB (nuclear factor-κB; −7.3 kcal/mol; 3 polar contacts). Overactivation of JAK2 may induce inflammatory responses and lead to the production of ROS, which secondarily increases protein glycation via the JAK2/signal transducer and activator of the transcription (STAT) pathway (Wang et al., 2022). ROS are also one of the activating factors of NF-κB. In endothelial cells, induction of NF-κB results in overexpression of vascular cell adhesion molecule-1 (VCAM-1) and thus increased adhesion of inflammatory cells to the vascular endothelium. This NF-κB-induced proliferation and inflammation results in vascular remodeling and chronic vascular invasion (Choi, 2023; Hoorzad et al., 2023). The main poze of verapamil showed three polar contacts with NF-κB through SER-220, SER-226, and deoxyadenosine (DA)-509. Verapamil can, therefore, directly inhibit the NF-κB transcription factor and also block its binding to the genetic material (Table 3; Figure 9).

## 4 Discussion

The prevalence of diabetes is increasing at an alarming rate worldwide. In 2019, 463 million people aged 20–79 suffered from diabetes, which is expected to rise to 578 million in 2030. Diabetes is also the third most common cause of death. 70%–80% of people with diabetes die from diabetes-related diseases, especially cardiovascular disorders (Candido et al., 2003; Glovaci et al., 2019). Despite many pathogenetic stimuli (diet, lifestyle, genetics, and immunology), glycoxidative stress is an essential factor linking glucose alternations and cardiovascular diseases (Peppa et al., 2002; Vlassara and Uribarri, 2004). Drugs that inhibit protein glycation could reduce the complications of diabetes and increase the life expectancy of patients.

One of the most commonly used drugs in cardiology is verapamil, a calcium channel blocker from the phenylalkylamine subgroup. The drug contains four major functional groups: tertiary amine, ether group, aromatic ring, as well as ketone group (Li and Shi, 2019; Fahie and Cassagnol, 2023) (Figure 1). The structure of verapamil is crucial to its therapeutic effect on the circulatory system. As the drug enters cells, it engages with L-type calcium channel proteins embedded in the cell membranes. Verapamil’s amine group, acting as a base, becomes protonated when it interacts with the channel protein. In this protonated form, the drug obstructs the calcium channel pore physically. This obstruction effectively prevents Ca^2+^ from passing through the channel during depolarization events. Simultaneously, verapamil’s aromatic ring contributes to steric hindrance, impeding the passage of Ca^2+^ even when the channel is open. Moreover, the drug interacts with specific voltage-sensitive regions of the calcium channel, stabilizing the channel in a closed conformation. This voltage-dependent inhibition further restricts Ca^2+^ influx into the cell. The combined effects of physical blockade, steric hindrance, and voltage-dependent inhibition substantially reduce intracellular Ca^2+^ levels (Li and Shi, 2019). In cardiac muscle cells, this reduction results in decreased contractility and a slower heart rate, which can be therapeutically beneficial in treating various arrhythmias. In smooth muscle cells of blood vessels, verapamil’s action induces relaxation, causing vasodilation and a consequent decrease in blood pressure (Guerrero and Martin, 1984; Fahie and Cassagnol, 2023). Due to the multiple actions of verapamil, it has been suggested that the drug also exhibits additional mechanisms of action. The structure of the drug (two aromatic unsaturated rings, four -OCH_3_ groups bonded with aromatic rings) indicates a potential antioxidant activity (Mak et al., 1992; Li and Shi, 2019).

We are the first to comprehensively evaluate the effect of verapamil on protein glycoxidation. For this purpose, we used the glycated/oxidized BSA models. Sugars (Glu, Fru, and Rib) and aldehyde (GO) were used as glycating factors, while ChT was applied as an oxidizing agent. We showed a relieving effect of verapamil on carbonyl/oxidative stress (Figures 3–7). In all models, biomarkers of protein glycation (↑APs, ↑βA, ↑AGEs), oxidation (↓TTs, ↑PCs, ↑AOPPs), as well as glycoxidation (↓TRY, ↑KN, ↑NFK, ↑DT) were ameliorated under the influence of verapamil. Verapamil, similarly to the protein glycation (aminoguanidine) and oxidation (Trolox) inhibitors, effectively counteracted the changes induced by glycating/oxidizing agents. The tested drug often restored them to BSA levels, and sometimes (βA in Glc and ChT, AOPPs in Rib, as well as TRY and NFK in Glc) even more than the baseline. Only TTs in Glc and Fru, as well as TRY in Fru, GO, and ChT, were not significantly enhanced by verapamil. Inhibiting protein glycoxidation is essential for maintaining insulin sensitivity, glucose metabolism, and overall vascular health. This process prevents the damaging effects of sugar-protein interactions, which can disrupt insulin signaling and lead to insulin resistance (Peppa et al., 2002; Vlassara and Palace, 2003; Vlassara and Uribarri, 2004; Aldini et al., 2013). Additionally, it can safeguard blood vessels from stiffening and narrowing due to the accumulation of AGEs, helping to maintain their normal function. Furthermore, inhibiting protein glycoxidation may slow the formation of atherosclerotic plaques in arteries, reducing the risk of cardiovascular complications (Lyons, 1993; Baynes and Thorpe, 2000; Aldini et al., 2013). This is pivotal in preventing diabetes-related issues and preserving cardiovascular wellbeing (Aldini et al., 2013).

In the body, not only plasma proteins undergo glycoxidation. Intracellular proteins such as hemoglobin and extracellular matrix proteins such as collagen are also vulnerable to this process (Vlassara and Palace, 2003; Shin et al., 2023). In animal studies, verapamil has been shown to reduce (in contrast to nifedipine and diltiazem) glycated hemoglobin (HbA1c) levels (Kaymaz et al., 1995). These results were also confirmed in clinical studies. Verapamil administration showed a significant reduction in HbA1c levels compared to patients receiving atenolol/chlortalidone, enalapril/hydrochlorothiazide, losartan/hydrochlorothiazide, trandolapril, as well as placebo (Cortassa et al., 1991; Ferrier et al., 1991; Fernández et al., 2001; Goicolea et al., 2002; Holzgreve et al., 2003). It is well known that HbA1c is the best biomarker of glycemic control used in diagnosing diabetes, as well as for assessing organ complications and the effectiveness of disease treatment (Sacks et al., 2023). Furthermore, it was found that verapamil may indirectly reduce collagen glycation in rats with type 2 diabetes. Wu et al. showed that valsartan exhibits a dose-dependent increase in the dynamic balance between osteogenesis and bone resorption in favor of the former. This is evidenced by increased levels of type I procollagen amino-terminal peptide (P1NP) and decreased levels of type I collagen C-terminal peptide (CTX-1) (markers of differentiation towards osteoblasts and osteoclasts, respectively) (Wu et al., 2022).

The antiglycative activity of verapamil may be due to its antioxidant properties. *In vivo*, verapamil has been shown to enhance the activity of vital antioxidant enzymes, including superoxide dismutase, glutathione peroxidase, and catalase, strengthening the cellular defenses against oxidative stress. Verapamil also inhibits lipid peroxidation and protein carbonylation, confirming its potential to mitigate oxidative damage to cellular biomolecules (Alican et al., 1994; Bukowska et al., 2008; Li et al., 2017; Jangholi et al., 2020). The antioxidant capacity of verapamil may partially explain the molecule’s chemical structure (Figure 1). Firstly, verapamil can provide resonance stabilization of trapped duo radicals to two aromatic unsaturated rings as a classic aromatic “chain-breaking” antioxidant. Secondly, verapamil also has four -OCH_3_ groups bonded with aromatic rings. The groups release electrons by which verapamil can neutralize chain-propagating lipid peroxy-radicals (Mak et al., 1992). It was shown that gallopamil, a close analog of verapamil, inhibits iron-dependent lipid peroxidation. Gallopamil differs from verapamil only in its additional methoxy group; therefore, the antioxidant properties of these drugs may be due to the chemical structure of phenylalkylamine derivatives. Indeed, it was shown that phenylalkylamine derivatives inhibit lipid peroxidation at several lower concentrations than dihydropyridine derivatives (Reddy et al., 1996).

The ability of verapamil to inhibit protein glycoxidation may also be explained by *in silico* analysis. In the molecular docking with a BSA macromolecule, verapamil formed polar bonds mainly with LYS (116, 132, 136), ARG (143), PHE (36), as well as TYR (139, 160). Of particular note is LYS-136, which forms a polar contact with the strongest binding affinity (Table 1; Supplementary Table S1; Figure 8). It is well known that basic (LYS, ARG, histidine [HIS]), aromatic (PHE, TYR, TRY), and sulfur-containing (cysteine [CYS], methionine [MET]) amino acids are particularly prone to glycoxidation (Lyons, 1993; Stadtman and Levine, 2003; Bader and Grune, 2006). The content of the individual amino acid groups in the structure of BSA is 17%, 8.4%, and 6.7%, respectively. Hence, the number of amino acids susceptible to glycation and oxidation accounts for about one-third of the total (32.1%). Our results indicate that verapamil may inhibit BSA modifications by binding to amino acids preferentially damaged by glycoxidation.

The second docking analysis was conducted between verapamil and glycosidases (αA, αG, and SI). Verapamil showed a high affinity for all of them. The role of αG in the assimilation of sugars is paramount. The active center of αG is mainly formed by aspartic acid (ASP)-322, glutamic acid (GLU)-340, glutamine (GLN)-465, ARG-494, asparagine (ASN)-495, and HIS-519 (Moorthy et al., 2012; Zhu et al., 2020). However, in our docking simulation, none of the above amino acids participate in binding to verapamil. We have shown that the most energetically favorable binding site is stabilized by as many as four polar interactions (three with SER-676 and one with GLY-651). It is the only one having an RMSD < 3 (Table 2; Supplementary Table S2; Figure 8). Thus, verapamil can bind to the αG outside its active center and incompetently inhibits the enzyme’s activity. Non-competitive/non-reversible inhibition could make the antidiabetic effect of verapamil more effective than acarbose (Whiteley, 2000). Acarbose is a reversible inhibitor of the αG enzyme, competing for its active center with complex carbohydrates (Martin and Montgomery, 1996; Hanefeld and Schaper, 2008).

The adverse effects of glycation in the body are due to the activation of the AGEs-RAGE signaling. Under these conditions, RAS, JNK, and mTOR are induced, which ultimately leads to NF-κB overexpression stimulating pro-inflammatory factors, such as cytokines, chemokines, NOX, and iNOS. JAK2-STAT and PI3K-PKB(Akt2) pathways are also activated, impairing cell proliferation/apoptosis and insulin transduction network. This promotes inflammation, oxidative stress, and structural changes in blood vessels, ultimately leading to complications such as atherosclerosis, nephropathy, retinopathy, and impaired vascular function. A molecular docking for AGE pathway proteins revealed that verapamil may owe its antiglycative properties *in vivo* (Pathomthongtaweechai and Chutipongtanate, 2020; Shen et al., 2020; Sindhuja et al., 2021). The drug presented a good docking ability to all tested ligands, starting at −4.6 kcal/mol for ATF4. An exceptionally high affinity was noted for JAK2 and NF-κB: −7.6 kcal/mol and −7.3 kcal/mol, respectively. The analysis did not reveal any polar contact for JAK2. In contrast, NF-κB bound to verapamil via SER-220, SER-226, as well as deoxyadenosine (DA)-509 (Table 3; Figure 9). Modulating the pathway proteins by verapamil may prevent adverse AGEs-mediated health effects such as insulin resistance, inflammation, or the generation of ROS, which aggravate protein glycation. Although further research on animal and human models is required, the tested drug could potentially counteract the development of diabetes and its complications.

The antiglycoxidant effect of verapamil may partially explain the drug’s multidirectional activity. In cardiology, verapamil effectively manages hypertension, angina, and arrhythmias, reducing myocardial oxygen demand and slowing electrical signals in the heart. It also has anti-ischemic effects, potentially safeguarding the heart during reduced blood flow (Krikler, 1986; Midtbø et al., 1986). In diabetology, verapamil may improve insulin sensitivity and glucose metabolism, benefiting type 2 diabetic patients (Fadda et al., 1989; Jia et al., 2022). Verapamil’s neuroprotective activity extends to migraine prevention, by which the drug is used in vasospastic disorders like Raynaud’s disease and Prinzmetal’s angina (Beller, 1989; Markley, 1991; Rirash et al., 2017). It is well known that both cardiovascular and neurological conditions have a glycoxidative etiology.

In conclusion, our results indicate verapamil’s antiglycative/antioxidant properties in *in vitro* and *in silico* models. The drug’s action is comparable to recognized substances protecting against oxidative and glycation modifications. In addition to the symptomatic treatment of cardiovascular disorders, verapamil can benefit patients with diabetes. Inhibition of carbonyl and oxidative stress by verapamil suggests a possible role for the drug not only in the treatment of cardiovascular symptoms but also in the prevention of diabetes-related diseases.

It should be mentioned that our study has several limitations. Firstly, the BSA model simplifies complex molecular interactions *in vivo*, making it difficult to transfer results to complex physiological contexts. Secondly, due to species-specific differences in glycation and oxidation patterns, direct extrapolation of outcomes to human systems could be compromised. Additionally, the influence of the extracellular matrix in the BSA environment might not faithfully mimic cellular microenvironments and signaling pathways. Temporal dynamics of glycation and oxidation processes might also be inadequately represented, impacting insights into their progression. Finally, verapamil undergoes extensive hepatic metabolism, where approximately 80% of the administered dose is subject to elimination through pre-systemic metabolism. The metabolites have no biological activity, except for norverapamil, which is attributed to up to 20% of the original drug’s pharmacological activity (Hamann et al., 1984; Zhou et al., 2009). Since norverapamil may have potential antiglycation effects, further studies should include not only verapamil but also its derivatives.

We realize that our research is preliminary. However, this is the first study to show a counteracting effect of verapamil on both carbonyl and oxidative stress. Given the increased incidence of diabetes and the lack of an antiglycating drug, studies in animal models and humans are needed to confirm verapamil’s antiglycative/antidiabetic mechanism of action. Therefore, our study provides a starting point for further research. Registration of a new indication for verapamil would be a therapeutic breakthrough, especially for people burdened with diabetes and cardiovascular problems.

## Data Availability

The original contributions presented in the study are included in the article/Supplementary Material. Further inquiries can be directed to the corresponding author.
