# Supplementary material for: Preliminary evaluation of the antiglycoxidant activity of verapamil using various in vitro and in silico biochemical/biophysical methods
Source: Front Pharmacol. 2023 Nov 28;14:1293295. doi: 10.3389/fphar.2023.1293295 (PMC10714945; doi:10.3389/fphar.2023.1293295)
Supplement: Supplementary file 1 [file Table1.DOCX]

**Table S1. Results of molecular docking simulation between verapamil and bovine serum albumin (BSA).**

ARG, arginine; LYS, lysine; PHE, phenylalanine; RMSD, root-mean-square deviations of atomic positions; THR, threonine; TYR, tyrosine.

| **Mode** | **Affinity (kcal/mol)** | **RMSD (lower bond)** | **RMSD (upper bond)** | **Amino acid residues** |
| --- | --- | --- | --- | --- |
| 1 | -7.6 | 0.000 | 0.000 | LYS-136 |
| 2 | -7.2 | 19.924 | 23.871 | TYR-160 |
| 3 | -6.3 | 4.933 | 8.982 | PHE-36 |
| 4 | -6.0 | 17.292 | 21.738 | LYS-132 |
| 5 | -6.0 | 2.406 | 8.514 | LYS-132 |
| 6 | -5.9 | 6.341 | 9.539 |  |
| 7 | -5.9 | 5.177 | 9.062 | LYS-116, TYR-160 |
| 8 | -5.9 | 14.229 | 18.912 | LYS-116, TYR-139, 2xARG-143 |
| 9 | -5.8 | 22.540 | 26.060 | THR-518 |

**Table S2. Results of molecular docking simulation between verapamil and α-glucosidase (αG).**

ARG, arginine; GLY, glycine; HIS, histidine; MET, methionine; PHE, phenylalanine; RMSD, root-mean-square deviations of atomic positions; SER, serine.

| **Mode** | **Affinity (kcal/mol)** | **RMSD (lower bond)** | **RMSD (upper bond)** | **Amino acid residues** |
| --- | --- | --- | --- | --- |
| 1 | -6.3 | 0.000 | 0.000 | GLY-651, 3xSER-676 |
| 2 | -6.1 | 33.631 | 36.969 |  |
| 3 | -5.9 | 34.422 | 38.399 | HIS-584 |
| 4 | -5.9 | 36.629 | 39.955 |  |
| 5 | -5.8 | 33.764 | 38.206 |  |
| 6 | -5.8 | 34.366 | 37.196 | ARG-585 |
| 7 | -5.8 | 34.681 | 38.723 | MET-363 |
| 8 | -5.8 | 30.873 | 33.827 | PHE-490, ARG-680 |
| 9 | -5.8 | 4.044 | 5.739 | ARG-281 |
